# Locus coeruleus toggles reciprocal prefrontal firing to reinstate fear

**DOI:** 10.1073/pnas.1814278116

**Published:** 2019-04-10

**Authors:** Thomas F. Giustino, Paul J. Fitzgerald, Reed L. Ressler, Stephen Maren

**Affiliations:** ^a^Department of Psychological and Brain Sciences, Texas A&M University, College Station, TX 77843-4235;; ^b^Institute for Neuroscience, Texas A&M University, College Station, TX 77843-3474

**Keywords:** extinction, relapse, locus coeruleus, norepinephrine, prefrontal cortex

## Abstract

The medial prefrontal cortex (mPFC) plays an essential role in regulating emotion, including inhibiting fear when danger has passed. The extinction of fear, however, is labile and a number of factors, including stress, cause extinguished fear to relapse. Here we show that fear relapse in rats limits single-unit activity among infralimbic (IL) neurons, which are critical for inhibiting fear responses, and facilitates activity in prelimbic (PL) neurons involved in fear expression. Pharmacogenetic activation of noradrenergic neurons in the locus coeruleus mimics this shift in reciprocal IL–PL spike firing, increases the expression of conditioned freezing behavior, and causes relapse of extinguished fear. Noradrenergic modulation of mPFC firing represents a mechanism for relapse and a potential target for therapeutic interventions to reduce pathological fear.

## Significance

Fear relapse represents a significant problem for individuals suffering from stress- and trauma-related disorders such as posttraumatic stress disorder. Here, we show that locus coeruleus norepinephrine activation produces fear relapse in rats. In addition, locus coeruleus activation inverts neuronal firing properties of the prelimbic and infralimbic cortices to drive this fear relapse. Elevated noradrenergic tone and subsequent changes in prefrontal firing properties represent a therapeutic target for combating fear relapse.

Learning to inhibit or extinguish fear when danger has passed is not only adaptive but also central to behavioral therapies for many psychiatric disorders. However, the extinction of fear is short-lived and relapse occurs under a variety of conditions, including psychological stress. Considerable data indicate that the prelimbic (PL) and infralimbic (IL) subdivisions of the medial prefrontal cortex (mPFC) serve to regulate the expression and inhibition of learned fear, respectively (1, 2). Projections from the locus coeruleus (LC) to the mPFC have a prominent role in stress-induced modulation of mPFC function (3–5). Moreover, noradrenergic transmission mediates stress-induced decreases in IL spike firing and impairments in extinction learning (6, 7). This work suggests that noradrenergic neurons in the LC may trigger relapse by altering mPFC firing dynamics to drive fear expression while weakening fear inhibition. Here, we explored this possibility using selective pharmacogenetic manipulation of LC noradrenergic neurons and mPFC single-unit recordings in rats undergoing relapse of extinguished fear.

## Results

**Fig. 1.**
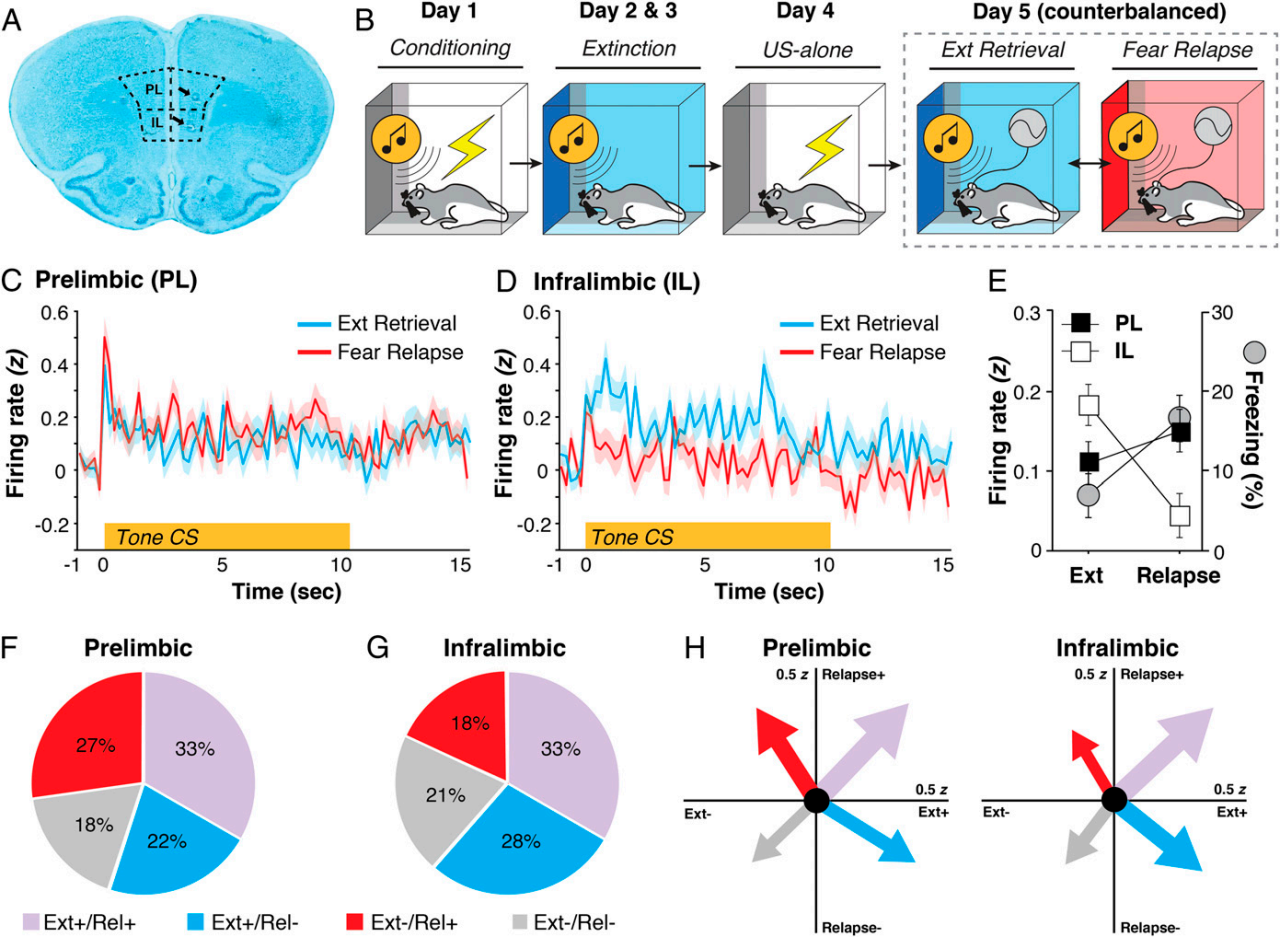
Extinction retrieval and fear relapse bidirectionally engage mPFC signaling. (*A*) Representative histology of electrode placements in PL and IL. (*B*) Schematized behavioral design. (*C* and *D*) CS-evoked firing from PL (*C*) and IL (*D*) neurons in retrieval and renewal. (*E*) Percentage of freezing (gray circles, mean ± SEM) across tests; freezing is overlaid with the 10-s summary of the CS-evoked firing responses. (*F* and *G*) Pie charts displaying the proportion of PL and IL neurons in one of four categories based on how neurons responded to presentation of the CS in both retrieval and relapse. (*H*) Vector plots depicting CS-evoked firing. The tips of the arrows point to the mean CS-evoked responding for a particular quadrant. The thickness of the arrow is proportional to the total number of neurons recorded in either PL or IL. Ext, extinction; Rel, relapse; US, unconditioned stimulus.

To characterize the neuronal correlates of extinction retrieval and fear relapse in the mPFC, we implanted animals with a single microelectrode array targeting both PL and IL and recorded single-unit activity using a within-subject behavioral design (Fig. 1 *A* and *B*). In this design, animals underwent standard auditory fear conditioning and extinction in distinct contexts; freezing behavior served as the index of fear (*SI Appendix*, Fig. S1). To facilitate the relapse of fear after extinction, animals received an unsignaled footshock unconditioned stimulus in the conditioning context to reinstate the fear memory (8). Single-unit recordings were then made in both the extinction context (where rats retrieved an extinction memory and expressed low levels of conditional freezing behavior) and a third distinct context (where rats retrieved a fear memory and expressed relatively higher levels of freezing behavior). Hence, this design combines two procedures that drive relapse of extinguished fear: reinstatement (reexposure to the unconditioned stimulus) and renewal (a context shift during retrieval testing); we refer this to as a “renewalment” procedure (8, 9). Importantly, the animals remained connected to the recording interface throughout these sessions so that the same mPFC neurons could be recorded during both retrieval tests (i.e., extinction retrieval and fear relapse).

During the test sessions, we recorded a total of 333 PL neurons and 288 IL neurons from 12 rats. Conditioned stimulus (CS)-evoked activity was normalized by calculating *z* scores for each post-CS bin (200 ms) relative to the firing rate in the 1-s pre-CS period; these *z* scores were averaged across the five CSs delivered during each test. As shown in Fig. 1, single-unit activity recorded in PL (Fig. 1*C*) and IL (Fig. 1*D*) neurons exhibited a reciprocal relationship in response to an identical auditory CS presented in the two distinct test contexts. Neurons in PL exhibited reliably higher CS-evoked firing in the relapse context relative to the extinction context, whereas the inverse was true among IL single units. This observation was confirmed in an ANOVA, which revealed a significant test context × brain region interaction on the average normalized firing rate in PL and IL to the test CSs [Fig. 1*E*; *F*(1, 629) = 11.73, *P* < 0.001]. The reciprocal firing in PL and IL mirrored CS-elicited freezing behavior (normalized to the 3-min pre-CS baseline), which was low in the extinction context and high in the relapse context [Fig. 1*E*; *F*(1, 11) = 6.05, *P* < 0.05].

Because we recorded the activity of the same prefrontal neurons during both retrieval tests, we were able to classify units according to four firing phenotypes defined by the direction of their CS-evoked response: excitatory (+, *z* > 0 for the 10-s CS averaged across five trials) or inhibitory (−, *z* < 0 for the 10-s CS averaged across five trials) in each of the two contexts (extinction or relapse). As shown in Fig. 1, these firing phenotypes were differently represented among the populations of neurons recorded in PL and IL (Fig. 1 *F* and *G*). A χ^2^ analysis revealed differences between PL and IL in terms of the proportion of neurons responding to the CS during extinction retrieval and fear relapse such that a larger proportion of PL neurons showed increased firing during relapse, whereas IL neurons were proportionately more active during extinction retrieval [χ^2^ (3) = 9.04, *P* < 0.05]. Fig. 1*H* depicts these data as population vectors that represent both the number of neurons in each phenotype (represented by arrow thickness) and the population mean of the average CS-evoked activity in each test context (indicated by the *x*,*y* coordinate of the arrow tip). These plots confirm that PL units fire preferentially in the relapse context, whereas IL units fire preferentially in the extinction context. Collectively, these data reveal that IL neurons showed more robust firing in response to the CS in the extinction context compared with the relapse context, whereas PL activity was higher relative to IL in the relapse context.

**Fig. 2.**
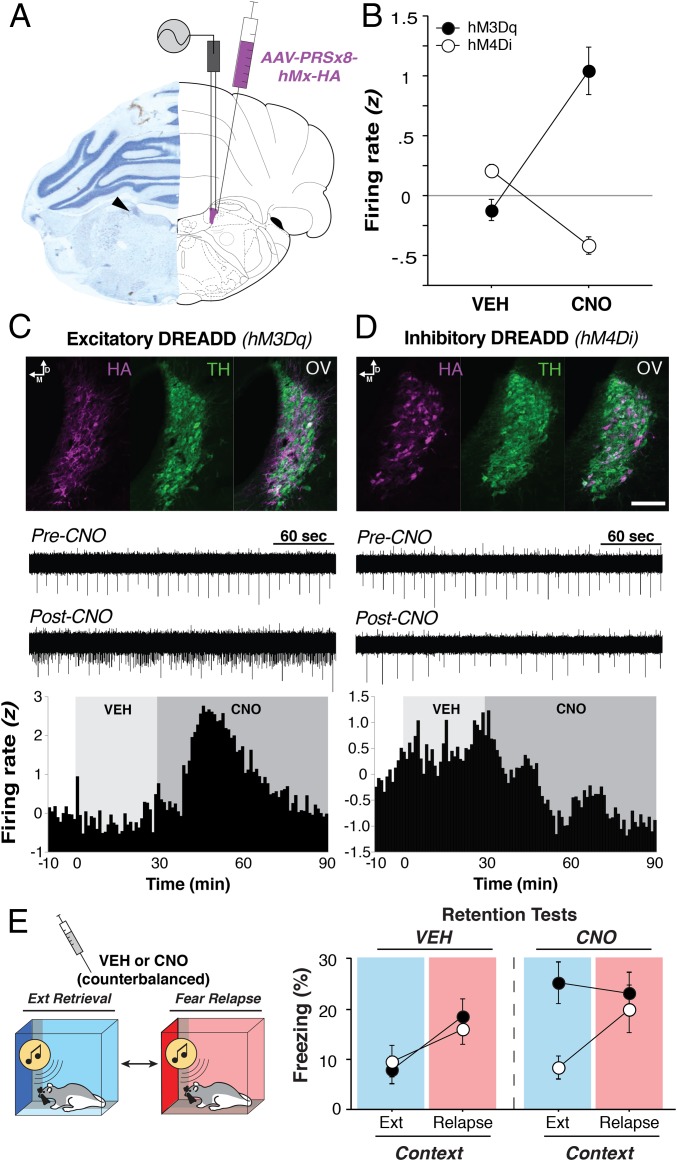
LC-specific DREADD functionality. (*A*) Representative microelectrode placement in the LC (*Left*), and a schematic (*Right*) indicating the placement of electrodes and DREADDs in the LC. (*B*) CNO administration bidirectionally regulates LC firing rates in animals expressing inhibitory (hM4Di) or excitatory (hM3Dq) DREADDs in the LC. (*C* and *D*, *Top*) Immunohistochemical localization of LC-DREADDs (HA, purple) in tyrosine hydroxylase-positive neurons (TH, green); OV, overlay. (Scale bar, 100 μm.) CNO administration produced robust increases (*C*) and decreases (*D*) in LC spike firing illustrated in both the raw recording traces (*Middle*) and the average firing rate (*Bottom*) of all neurons recorded. (*E*, *Right*) Percentage of freezing (mean ± SEM) for each group across days. CNO produced fear relapse in the hM3Dq group (black circles), whereas LC inhibition via hM4Di (white circles) had minimal effects on freezing in extinction retrieval and fear relapse. Background colors within the freezing graphs correspond to each session’s context (*Left*), with blue indicating extinction retrieval and red indicating fear relapse.

Given that noradrenergic transmission mediates stress-induced decreases in IL spike firing and impairments in extinction learning (6, 7), we hypothesized that noradrenergic neurons in the LC would drive fear relapse. To selectively target noradrenergic LC neurons (Fig. 2*A*), we used custom excitatory and inhibitory designer receptors exclusively activated by designer drugs (DREADDs) whose receptor expression is under control of the synthetic dopamine-β-hydroxylase PRSx8 promoter (10). To confirm the in vivo functional efficacy of these LC-specific DREADDs, animals expressing either AAV9-PRSx8-hM3Dq-HA (Fig. 2*C*; an excitatory DREADD) or AAV9-PRSx8-hM4Di-HA (Fig. 2*D*; an inhibitory DREADD) were anesthetized and implanted with a recording array for acute LC recordings. We used a within-subject design to record the activity of the same neurons (*n* = 54, hM3Dq; *n* = 100, hM4Di) in response to both vehicle (VEH) and clozapine N*-*oxide (CNO, 3 mg/kg, i.p.). After a 10-min baseline period, rats were injected with VEH, and recording continued for 30 min, upon which rats were then injected with CNO, followed by recording for an additional 60 min to observe the changes in firing rate.

The spontaneous baseline LC firing rates before drug administration were as follows (mean ± SEM): hM3Dq (2.09 ± 0.12 Hz) and hM4Di (1.83 ± 0.09 Hz). To assess CNO-induced changes in spike firing, we normalized the postinjection firing rates (60-s bins across the entire 100-min recording session) to the 10-min baseline period. As shown in Fig. 2 *B–D*, CNO induced statistically reliable changes in average LC firing rate [Fig. 2*B*; drug × virus interaction, *F*(1, 122) = 117.7, *P* < 0.0001]. It significantly increased LC firing in 76% (41/54) of the neurons recorded in hM3Dq-expressing rats [Fig. 2*C*; main effect of time, *F*(69, 3,657) = 22.00, *P* < 0.0001] and decreased LC firing in 65% (65/100) of the neurons recorded in hM4Di-expressing animals [Fig. 2*D*; main effect of time, *F*(69, 6,831) = 15.11, *P* < 0.0001].

After confirming the functional efficacy of the LC DREADDs, we next determined whether manipulating LC activity would influence extinction retrieval and fear relapse in the within-subject renewalment design. As shown in Fig. 2*E*, VEH-treated rats expressing inhibitory or excitatory LC DREADDs showed low levels of CS-elicited freezing (normalized to baseline) in the extinction context, but a marked increase in freezing in the relapse context. Interestingly, pharmacogenetic activation of noradrenergic neurons in the LC was sufficient to induce fear relapse; CNO administration in hM3Dq-expressing rats dramatically increased freezing in the extinction context. Inhibiting LC activity, however, did not prevent fear relapse [drug × context × virus interaction, *F*(1, 40) = 5.17, *P* < 0.05]. This is not surprising insofar as relapse associated with a context shift is independent of contextual fear (11). In addition to increasing freezing to the CS, CNO administration produced significant increases in freezing before delivery of the CS (during the baseline period) in animals expressing hM3Dq in the LC (*SI Appendix*, Fig. S2). Note that this increase in baseline freezing was independent of the relapse effect, which was manifest as an increase in CS-evoked freezing normalized to the elevated baseline. The observation that LC activation increases freezing behavior is consistent with recent work showing that LC activation induces anxiety-like behavior (12, 13). These results reveal that pharmacogenetic activation of noradrenergic LC neurons promotes the relapse of extinguished fear.

**Fig. 3.**
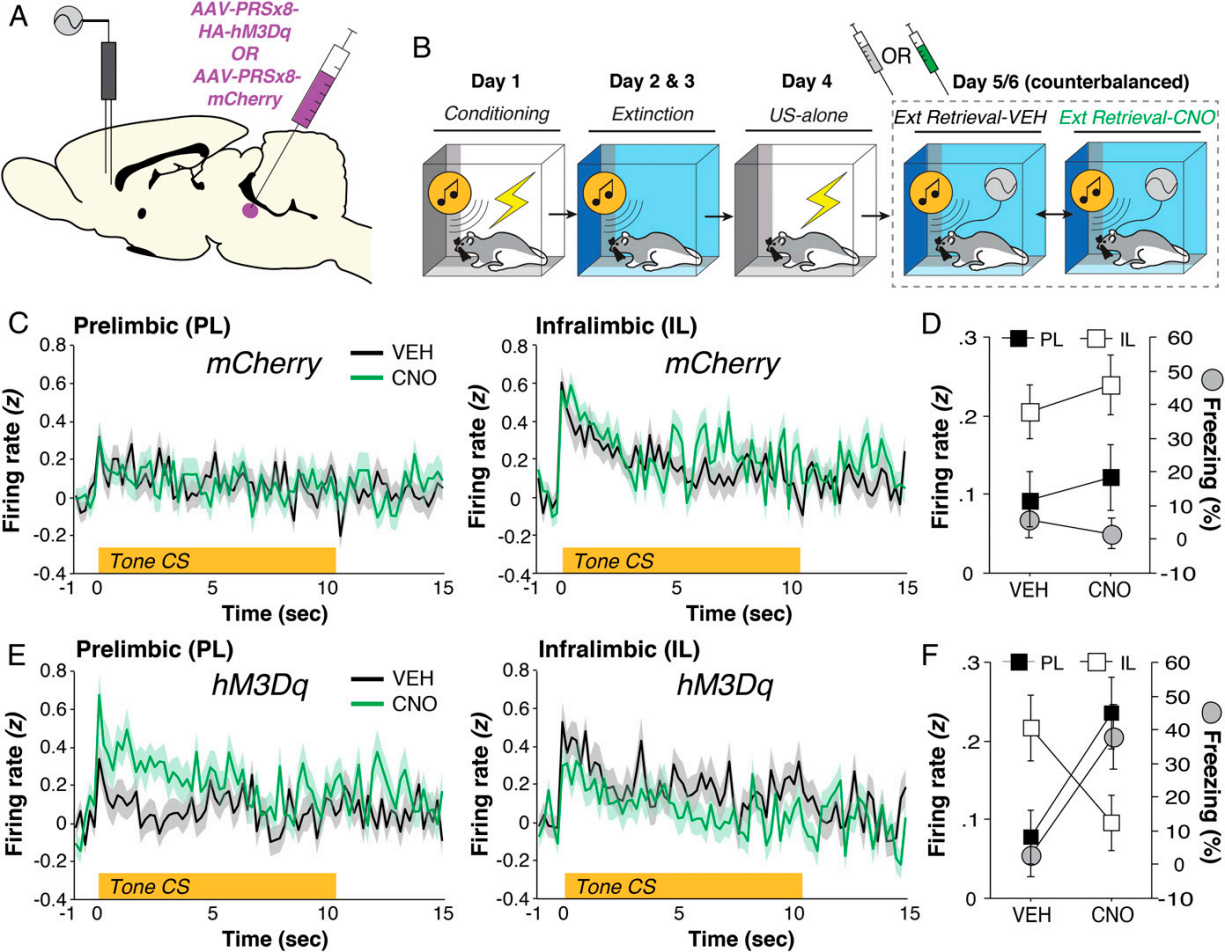
LC-NE drives PL CS-evoked activity and fear relapse. (*A*) Schematic representation of experimental approach. (*B*) Schematized behavioral design. (*C*) CS-evoked responses in PL (*Left*) and IL (*Right*) during extinction retrieval after either VEH or CNO administration in animals expressing the blank mCherry vector. (*D*) Percentage of freezing (gray circles, mean ± SEM) across test days; freezing is overlaid with the 10-s summary of the CS-evoked firing responses for each brain region. (*E*) CS-evoked responses in PL (*Left*) and IL (*Right*) in animals expressing hM3Dq in the LC after either VEH or CNO administration. (*F*) Percentage of freezing (gray circles, mean ± SEM) across test days; freezing is overlaid with the 10-s summary of the CS-evoked firing responses for each brain region. Ext, extinction; US, unconditioned stimulus.

The relapse of extinguished fear is associated with the suppression of activity in IL-amygdala circuits involved in the inhibition of fear (14, 15). Based on previous work showing that noradrenergic transmission mediates stress-induced reductions in IL firing (6), we hypothesized that LC-driven fear relapse is mediated by a suppression of IL spike firing in the mPFC. To test this hypothesis, microelectrode-implanted rats (targeting PL and IL) expressing either AAV9-PRSx8-hM3Dq-HA or an AAV9-PRSx8-mCherry control virus (*SI Appendix*, Fig. S3) in the LC (Fig. 3*A*) underwent fear conditioning, extinction, and retrieval tests in the extinction context after either VEH or CNO administration (Fig. 3*B* and *SI Appendix*, Fig. S4). The number of neurons recorded in each group and brain area are as follows: VEH mCherry [PL, *n* = 160; IL, *n* = 135]; CNO mCherry [PL, *n* = 134; IL, *n* = 136]; VEH hM3Dq [PL, *n* = 131; IL, *n* = 105]; CNO hM3Dq [PL, *n* = 105; IL, *n* = 116]. As shown in Fig. 3 *C–F*, pharmacogenetic activation of noradrenergic LC neurons increased CS-evoked spike firing in PL and suppressed that in IL. In other words, LC activation shifted mPFC firing from a low-fear (IL > PL) to a high-fear profile (PL > IL) [Fig. 3 *D* and *F*; drug × virus × region interaction, *F*(1, 1,010) = 6.80, *P* < 0.01] and drove fear relapse [Fig. 3 *D* and *F*, gray circles; *F*(1, 11) = 7.93, *P* < 0.05]. Collectively, these results reveal that LC activation toggles reciprocal firing in the mPFC by decreasing CS-evoked spike firing in IL on the one hand, while increasing PL spike firing on the other. This inversion of extinction-related mPFC firing results in the relapse of extinguished fear.

**Fig. 4.**
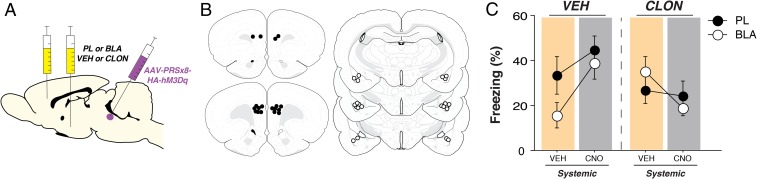
Local infusions of clonidine in either the PL or BLA block CNO-induced fear relapse to a previously extinguished CS. (*A*) Schematic representation of the experimental approach. (*B*) Schematic histology displaying location of cannula tips in either the PL or BLA. (*C*) Percentage of freezing (mean ± SEM) across test days split by brain region. Data are normalized by subtracting the five-trial CS-evoked averages from the 3-min stimulus-free baseline period. Background colors depict systemic injections (orange, vehicle; gray, CNO).

Although the previous data strongly implicate a role for LC-norepinephrine (NE) modulation of mPFC CS-evoked firing, they do not causally implicate LC projections to mPFC in the observed neural and behavioral changes. To address this question, we sought to determine whether the propensity of CNO-induced LC activation to cause fear relapse could be antagonized by pharmacologically reducing NE release in the PL with intracranial infusions of clonidine, an alpha2-adrenoceptor agonist. Because it has been suggested that LC actions in the basolateral amygdala (BLA) might also come to influence the mPFC (3–5), we also included animals in which we reduced NE release in the amygdala. To this end, animals expressing AAV9-PRSx8-hM3Dq-HA were implanted with bilateral cannula targeting either the PL or BLA to examine if CNO-induced LC activation mediates its behavioral effect via one of these targets. Animals underwent fear conditioning, extinction, and retrieval tests in the extinction context (*SI Appendix*, Fig. S5). Using a within-subject design (Fig. 4*A*), animals received intracranial infusions of either vehicle or clonidine (order counterbalanced) in either the PL or BLA (Fig. 4*B*). After intracranial infusions, animals were injected with systemic VEH or CNO (order counterbalanced) and, ∼20 min later, underwent extinction retrieval as in the previous experiments. In this design, each rat underwent four separate extinction test sessions. As shown in Fig. 4*C*, intracranial clonidine infusions reduced CNO-induced increases in CS-evoked freezing relative to VEH controls [systemic drug × intracranial drug interaction, *F*(1,18) = 8.04, *P* < 0.05]. This effect was similar whether clonidine was infused in PL or BLA. These data reveal that NE release in both the PL and BLA mediates the behavioral effects of pharmacogenetic activation of the LC. This suggests that NE release in the BLA might drive, at least in part, the neuronal correlates of fear relapse observed in the PL.

## Discussion

Collectively, these experiments uncover a role for LC modulation of mPFC spike firing in the relapse of extinguished fear. Specifically, we demonstrate that DREADD-induced increases in LC firing toggle reciprocal spike firing in the mPFC and drive relapse of extinguished fear. In particular, LC activation increased CS-evoked responding in PL, while decreasing that in IL, an inversion of the IL-dominated firing observed after extinction (1, 11, 16). These data reveal that noradrenergic neurons in the LC modulate mPFC signaling to induce neuronal firing signatures associated with high fear states, which in turn drives relapse.

The current data confirm and extend previous work revealing dissociable roles of PL and IL in conditioned fear (17–23). We demonstrate that CS-evoked firing in IL is most pronounced in the extinction context (where fear is low), whereas it is reliably lower in the relapse context (where fear is high). In contrast, this pattern is inverted in PL, where CS-evoked spike firing is relatively higher in the relapse compared with the extinction context. Although it is well established that PL and IL firing correlate with high and low fear states respectively, the neural circuitry and transmitter systems driving these differences are relatively unknown. We now demonstrate a critical role for the LC-NE system in driving differential responses in PL and IL: Pharmacogenetic activation of the LC increased PL firing (relative to IL) and caused the relapse of extinguished fear. One possibility is that direct LC→PL projections excite PL pyramidal cells, which in turn inhibit the IL; others have shown that PL→IL connections can influence freezing behavior (24). A second possibility is that these differences are driven by indirect pathways from the LC to the mPFC via the amygdala. Past work has shown that BLA projections to PL and IL mediate high and low fear states, respectively (25). The fact that reducing NE release in either the PL or BLA prevented LC-induced fear relapse suggests that it may be a combination of these pathways that mediates the effects of LC activation on fear.

The LC-NE system has been widely studied in the context of stressor- and trauma-related disorders such as posttraumatic stress disorder (PTSD) (3, 26–28). For example, prazosin, an alpha1-adrenoceptor antagonist, has had some success in reducing nightmares associated with PTSD (29–32, but see ref. 33). In addition, guanfacine and clonidine (alpha2-adrenoceptor agonists) as well as propranolol (a beta1,2-adrenoceptor antagonist) have shown promise in alleviating PTSD symptomatology (3, 5, 34). However, here, we show no effect of pharmacogenetic inhibition of the LC on either extinction retrieval or fear relapse. This suggests that noradrenergic antagonists such as propranolol might not be effective in reducing the acute relapse of extinguished fear. Of course, it is possible that the degree of inhibition we obtained with inhibitory DREADDs was not sufficient to prevent NE release in LC terminals in the forebrain.

Overall, these data have important clinical implications insofar as elevated NE levels are observed in patients with PTSD and have been argued to underlie, at least in part, the pathophysiology of this disorder (34–37). Consistent with this, noradrenergic transmission causes stress-induced decreases in IL firing and impairs extinction learning (1, 2, 4), which may underlie extinction-learning deficits in individuals suffering from PTSD (4, 5, 34, 38, 39). We now show that noradrenergic neurons in the LC influence mPFC spike firing to drive the return of fear once it has been extinguished. As such, noradrenergic tone along with mPFC activity may serve as a reliable biomarker to predict fear relapse. Moreover, pharmacotherapeutic interventions that moderate LC hyperactivity in PTSD might be particularly effective in promoting long-lasting extinction learning and preventing its relapse once learned (6, 7, 34).

## Materials and Methods

A detailed description of materials and methods can be found in *SI Appendix*, *Materials and Methods*. All procedures were conducted at Texas A&M University and performed in strict accordance with the guidelines and regulations set forth by the National Institutes of Health and the Texas A&M University, with full approval from its Institutional Review Board and Animal Care and Use Committee.

### Behavioral Procedures.

Briefly, behavioral experiments were conducted as previously described; each context was made distinct by varying features of the chamber, including (but not limited to) olfactory cues and lighting conditions (6, 7). Rats underwent auditory fear conditioning, extinction, and within-subject retrieval testing in either the extinction context or another relapse context.

### Electrophysiology.

Extracellular single-unit activity was recorded with a multichannel neurophysiological system (OmniPlex; Plexon) in behaving animals during the retrieval tests. Wideband signals were amplified, digitized, and then saved on a personal computer for offline sorting and analysis as previously described (6).

### LC-Specific DREADDs.

Viral constructs were obtained from the University of Pennsylvania Vector Core. The constructs used throughout the paper are as follows: AAV9-PRSx8-hM3Dq-HA, AAV9-PRSx8-hM4Di-HA, and AAV9-PRSx8-mCherry. The PRSx8 promoter restricts expression to noradrenergic neurons (10).

### Histology.

After completion of the experiment, the rats were overdosed with pentobarbital. Rats were then perfused transcardially with 0.9% saline, followed by 10% formalin. Brains were extracted from the skull and postfixed in a 10% formalin solution for 24 h, followed by a 30% sucrose solution, in which they remained for a minimum of 48 h. Coronal brain sections of mPFC or BLA (40-µm thickness) were cut on a cryostat (−20 °C, Leica Microsystems), mounted on subbed microscope slides, and stained with thionin (0.25%) to visualize electrode and cannula placements. LC viral expression was confirmed using conventional immunohistochemical techniques.

### Statistics.

We analyzed the data with conventional parametric statistics (StatView, SAS Institute). Two-way ANOVA and repeated-measures ANOVA were used to assess general main effects and interactions (α = 0.05). Data are represented as mean ± SEM.

## Supplementary Material

Supplementary File
