# Supplementary material for: Locus coeruleus toggles reciprocal prefrontal firing to reinstate fear
Source: Proc Natl Acad Sci U S A. 2019 Apr 10;116(17):8570–5. doi: 10.1073/pnas.1814278116 (PMC6486780; doi:10.1073/pnas.1814278116)
Supplement: Supplementary File [file pnas.1814278116.sapp.pdf]

SI Appendix

SI Materials and Methods

Subjects

Eighty-seven experimentally naïve adult male Long-Evans Blue Spruce rats (weighing 200-224 g; 50-57 days old) were obtained from a commercial supplier (Envigo, Indianapolis, IN). Upon arrival and throughout the experiments, rats were individually housed in cages within a humidity- and temperature-controlled vivarium, and kept on a 14:10 hr light/dark cycle (lights on at 7 am) with ad libitum access to food and water. All experiments were conducted in the daytime during the light phase. Rats were handled for ~30 seconds a day for 5 days to habituate them to the experimenter before any behavioral testing or surgical procedures were carried out. All procedures were conducted at Texas A&M University and were performed in strict accordance with the guidelines and regulations set forth by the National Institutes of Health and Texas A&M University with full approval from its Animal Care and Use Committee.

Experimental Procedures:

Locus coeruleus-specific DREADDs and behavioral procedures

Rats were bilaterally infused with a locus coeruleus (LC)-specific DREADD (Designer Receptors Exclusively Activated by Designer Drugs). For viral infusion surgery, rats were anesthetized with isoflurane (5% induction, 2% maintenance) and placed in a stereotaxic apparatus (Kopf Instruments, Tujunga, CA). The scalp was incised and retracted, the head was tilted 15 degrees downward such that bregma skull surface was 2 mm below intersectional lambda skull surface in the horizontal plane. The skull overlying the left and right hemispheres of LC was removed. Both hemispheres of LC were then separately infused with either the LC-specific excitatory DREADD (AAV9-PRSx8-hM3Dq-HA), with an inhibitory one (AAV9-PRSx8-hM4Di-HA), or a blank control virus (AAV9-PRSx8-mCherry), using a hypodermic injector (Small Parts/Amazon, Seattle, WA) that was coupled to a Legato 101 infusion pump (KD Scientific, Holliston, MA) and 10 µl syringe (Hamilton Company, Reno, NV) using polyethylene tubing (Braintree Scientific, Braintree, MA). The coordinates for each infusion (relative to intersectional lambda skull surface) were as follows: AP: -3.8, ML: +/-1.4. Since these were LC-specific DREADDs(1) and the depth of the LC is variable across rats, virus was infused at three separate depths from lambda skull surface (first infusion: -7.0, second: -6.5, third: -6.0), 0.5 µl was infused at each depth. The infusion rate was 0.25 µl/min, and after the third infusion the injector was left in the brain for an additional 5 min to allow the virus to diffuse more effectively at the infusion site. At least two weeks were allowed for recovery, and for the virus to express in the LC, before experiments began.

A modified rodent behavioral chamber (30x24x21 cm, Med Associates, St. Albans, VT) enclosed in a sound-attenuating cabinet was used for most days (i.e., the two extinction sessions, two dual retrieval-renewal tests) of these behavioral experiments. This chamber was modified to allow for freely moving electrophysiological recordings as well (described later). The chamber comprised two aluminum side walls, a Plexiglas rear wall, a hinged Plexiglas door, and an open top. The grid floor consisted of 19 stainless steel rods (4 mm diameter) spaced 1.5 cm apart (center-to-center). A loudspeaker attached to the outside of a grating in one aluminum wall was used to play auditory tones. Locomotor activity of the rat was transduced by a load-cell under the floor of the chamber, and the output of the load-cell was recorded by an OmniPlex recording system (Plexon, Dallas, TX). Thus, all behavioral activity (and neural activity, for the freely

moving recordings) was recorded automatically with this system. A separate but very similar (i.e., enclosed top) behavioral chamber (context A), located in an adjacent room, was used for fear conditioning and to deliver a reminder shock in a later session (see below); having a separate room for context A helped reduce fear generalization across contexts. No electrophysiological recordings took place in this chamber, and locomotor activity was recorded automatically using a computerized load-cell system. The rods comprising the grid floor were connected to a shock source and solid-state grid scrambler (Med Associates) for the delivery of footshocks.

On Day 1 of behavioral testing, each rat was individually fear conditioned in context A. In this procedure, the rat was transported to the room in a white plastic box and placed in the behavioral chamber. The chamber had been cleaned with 70% ethanol to provide a distinct olfactory cue, and a metal pan containing a thin layer of the same solution had been placed under the grid floor. The room was illuminated with white ambient lights, although the chamber house light was off and the sound attenuating chamber doors were closed, with a small window in one of the doors to allow some light in. A fan mounted within one wall of the sound-attenuating chamber was also turned on to provide constant, ambient background noise (context A). After a 3-min stimulus-free baseline period, the animal received three auditory tone-footshock pairings. The tones (conditioned stimuli; CS) were 10 sec, 80 dB, 2 kHz; the shocks (unconditioned stimuli; US) were 2 sec and 1 mA, where shock onset occurred at tone offset. There was a 1-min inter-trial interval (ITI) between shocks. The behavioral session continued for 1 min after the final shock, and then the rat was returned to its home cage.

On Day 2, the first of two fear extinction sessions took place, in the recording room adjacent to where fear conditioning was administered. The rat was transported to the room in a black plastic box and placed in the behavioral chamber. The chamber had been cleaned with 3% acetic acid to provide a distinct olfactory cue, a black plastic pan containing a thin layer of the same solution had been placed under the grid floor, the grid floor was covered with a transparent rubber mat, the back wall was covered with alternating black and white stripes, and the room was illuminated with ambient red lights (context B). After a 3-min stimulus-free baseline period, the animal was presented with 45 tone-alone trials (30-sec ITI); the rat remained in the chamber for 3 min after the final tone, and movement was recorded automatically throughout the session. Day 3 consisted of a second extinction session, identical to that of Day 2. On Day 4, the rat received an unsignaled (i.e., no tone was presented) reminder shock in the conditioning chamber (context A). After a 3-min baseline, this weaker shock (0.5 mA, 2 sec) was delivered, followed by a 3-min stimulus-free period. As in the Day 1 session, movement was recorded automatically.

On Day 5 (and Day 6 for experiments that had 2 test days), the rat received a dual retrieval-renewal test. For animals that received LC DREADDs and an mPFC array, the DREADD ligand, clozapine N-oxide (CNO; 3 mg/kg, i.p.) or a vehicle (VEH; 2.5% dimethyl sulfoxide [DMSO] in distilled water) injection was given 30 min before the start of testing. In this within-subjects design, each rat received CNO one day and VEH the other day of testing, with drug sequence counterbalanced across rats. For a given rat, whether retrieval or renewal came first within the test was held constant across Days 5 and 6, and this was also counterbalanced across rats. The following is a description of the behavioral procedure if retrieval took place first. The rat was transported to the recording room in a black plastic box and placed in the behavioral chamber (context B). After a 3-min stimulus-free baseline period, the animal was presented with 5 tone-alone trials (30-sec ITI) and remained in the chamber for 10 min after the final tone. The rat was then immediately placed in a large white plastic bucket with a layer of bedding in the bottom. The contextual cues were then rapidly (within approximately 5 min) altered as follows to prepare for

the fear renewal session: 1% ammonium scent, white plastic pan beneath the grid floor, no rubber mat, no striped walls, white ambient lighting (context C). The renewal protocol was the same as in retrieval: 3-min baseline, 5 tone-alone trials, 10-min stimulus-free period after the last tone. The rat was then returned to its home cage.

Electrophysiological characterization of LC DREAADs

Rats were anesthetized with isoflurane (5% induction, 2% maintenance) and placed in a stereotaxic apparatus (Kopf Instruments). The scalp was incised and retracted, the head was tilted downward 15 degrees as described above, and the skull was cleaned to allow for the acute insertion of the electrode array into LC. The microelectrode array (Innovative Neurophysiology, Durham, NC) comprised 16, 10.5 mm long wires. This 4 x 4 wire array had 200 µm center-to-center spacing of adjacent wires. Each wire was 50 µm in diameter and the conductor was tungsten. Using the same coordinates as described above for viral infusion in LC, the array was slowly lowered to a depth of 6-7 mm, while the experimenters listened to an audio output of the neural activity through our Plexon recording system. After lowering the electrode array into LC, we allowed the signal to stabilize for at least 30 min, and then the recording session began. After a 10 min baseline period, the rat was gently injected (i.p.) with VEH (the Plexon file was briefly paused for this), followed 30 min later by CNO (Plexon file again briefly paused); the recording session continued for 60 more minutes. Immediately afterward, the rat was deeply anesthetized with pentobarbital and perfused transcardially (see Histology section below).

Extracellular single-unit activity was recorded with a multichannel neurophysiological recording system (OmniPlex, Plexon, Dallas, TX). Wideband signals recorded on each channel were referenced to one of the recording wires (resulting in a maximum of 15 channels of activity per rat), amplified (8,000x), digitized (40 kHz sampling rate), and saved on a PC for offline sorting and analysis. The recording reference wire we chose for each session was selected to optimize the quality of the recordings. After high-pass filtering the signal at 600 Hz, we sorted waveforms manually using 2-dimensional principal component analysis (Offline Sorter, Plexon). Only well-isolated units were used in our analysis. If two units with similar waveforms and identical time stamps for their action potentials appeared on adjacent electrodes, we only used one unit. We then imported sorted waveforms and their timestamps to NeuroExplorer (Nex Technologies, Madison, AL) for further analysis.

***In vivo* electrophysiology in freely moving rats**

For surgeries in the animals that were implanted with a chronic microelectrode array targeting the mPFC, the rat was anesthetized with isoflurane and secured in a stereotaxic apparatus (Kopf Instruments). Some of these animals also received bilateral infusions of LC virus prior to mPFC array implantation: the scalp was incised and retracted, the head was tilted 15 degrees downward, and either the excitatory or blank control virus was bilaterally infused into LC as described above. The head was then returned to horizontally level for the rest of the surgery. The following description applies to all of the mPFC recording rats. Next, three-five burr holes were drilled for anchor screws. The region of the skull overlying mPFC was removed to allow for microelectrode implantation. The animal was then implanted with a 16-channel microelectrode array (Innovative Neurophysiology) targeting both PL (8 wires) and IL (8 wires) in the right hemisphere. This 2 x 8 wire microarray was constructed from two rows of 50 µm diameter tungsten wires of two different lengths (PL, 6.9 mm; IL, 8.0 mm; see below for dorsal-ventral coordinates); the wires in each row and the rows themselves were spaced 200 µm apart (center-to-center). The array was positioned

with its long axis parallel to the anteroposterior plane. Coordinates for the centermost wires of the array were (relative to bregma skull surface): +2.7 mm AP, +0.55 mm ML, -4.0 mm DV for PL; and +2.7 mm AP, +0.35 mm ML, -5.1 mm DV for IL. The array was secured to the skull with dental acrylic and at least two weeks were allowed for recovery and viral expression before *in vivo* recordings began.

As described above, a standard rodent behavioral chamber (Med Associates) enclosed in a sound-attenuating cabinet was modified to allow for electrophysiological recordings. Neural activity and locomotor output was recorded automatically by an OmniPlex recording system (Plexon). The behavioral procedure for these rats took place across five (mPFC only recording rats) or six (LC virus + mPFC recording rats) consecutive days, and was the same as described above except that neural recordings were obtained on the two test days (Days 5 and 6). For the recording days, the rat was connected to a headstage with a flexible cable (Plexon) before the session began, and then placed in the recording chamber. Immediately after the session, the rat was unplugged from the headstage and returned to its home cage. For the initial mPFC recording experiment (i.e., rats that did not receive LC DREADDs), the rat remained plugged in between the retrieval and renewal sessions while placed in the large white plastic bucket with a layer of bedding in the bottom, while the contexts were rapidly changed. For the LC virus + mPFC recording experiment, rats only underwent a retrieval session on both Day 5 and 6 following either VEH or CNO administration. Neural data were collected and analyzed as described above for the anesthetized recordings. The recording reference electrode we chose was typically one of the eight wires located in PL. The analysis of neural activity focused on CS-evoked activity during the Day 5 and 6 test sessions. For analysis of the CS-evoked activity, firing rate was binned in 200-msec increments around the time of the tones for individual neurons, and the evoked responses were z-score normalized to the 1-sec period prior to tone onset, averaged across the five tones.

Cannula implantation for intracranial infusions

For surgeries in animals that were implanted with bilateral cannula targeting PL or BLA, rats were anesthetized with isoflurane and secured in a stereotaxic apparatus (Kopf Instruments). They first received bilateral infusions of the AAV9-PRSx8-hM3Dq-HA virus in the LC as described above. After viral infusions, the head was placed in the horizontal plane for implantation of bilateral guide cannulae in the PL (8mm, 26 gauge; Plastics One; AP: + 2.7, ML: +/- 2.0 (insertion point), DV: -3.8 at a 20-degree angle) or the BLA (10mm, 26 gauge; Plastics One; AP: - 2.9, ML: +/- 4.8, DV: -8.55); all coordinates relative to bregma. Three-five burr holes were drilled in the skull for jeweler's screws and dental acrylic was applied to the skull to secure the cannula in place. Dummy cannula (33 gauge) were placed into the guide cannula upon completion of the surgery.

After recovery from surgery, intracranial infusions were performed as previously described (2). Briefly, rats were transported to the infusion room in 5-gallon buckets. Dummies were removed and stainless steel injectors (33 gauge) were inserted into the guide cannula for local infusion of drug. All infusions were made using Hamilton syringes mounted in an infusion pump and connected to the injectors with polyethylene tubing. The infusions were made approximately 25-30 minutes before the behavioral procedures. Clonidine hydrochloride (Sigma-Aldrich, St. Louis, MO) was dissolved in saline (5.83 ug/ul) and infused into either PL or BLA (0.3 ul/side at 0.25 ul/min); this dose (1.75 ug/side) has previously been shown to reduce conditioned freezing behavior (3, 4). Injectors remained in place for 1 min after the infusion to allow for drug diffusion. After the infusions, clean dummies were inserted in the guide cannulas and the rats were injected

with either systemic VEH or CNO in a counterbalanced fashion. Extinction retrieval tests commenced approximately 20 min following the systemic injections.

Histology

After completion of the experiment, the rats were overdosed with pentobarbital. For rats implanted with an mPFC array, electrolytic lesions were created by passing electrical current (80 µA, 10 sec; A365 stimulus isolator, World Precision Instruments, Sarasota, FL) through six of the recording wires (anterior, middle, posterior wires in both PL and IL). Rats were then perfused transcardially with 0.9% saline followed by 10% formalin. Brains were extracted from the skull and post-fixed in a 10% formalin solution for 24 hours, followed by a 30% sucrose solution, where they remained for a minimum of 48 hours. Coronal brain sections of the mPFC or BLA (40 µm thickness) were cut on a cryostat (-20° C, Leica Microsystems, Buffalo Grove, IL), mounted on subbed microscope slides, and stained with thionin (0.25%) to visualize electrode or cannula placements.

To visualize LC viral expression using immunohistochemistry, the following steps were carried out. First, brains were coronally sectioned (40 µm thickness) with a cryostat and stored in a 0.01% sodium azide solution until further processing. Sections were blocked in PBS with 0.1% Triton X-100 (TX) and 3% normal donkey serum (NDS, 2 ml/well) for one hour. All steps occurred in this PBS-TX-NDS solution at room temperature. Sections were then incubated in primary antibodies (mouse anti-tyrosine hydroxylase (TH) [1:2000] and rabbit anti-HA [1:1000]) for 24 hours. Sections were then rinsed three times (10 min each). Sections were then incubated in secondary antibodies (donkey anti-mouse Alexa Fluor 488 [1:500; for TH] and donkey anti-rabbit Alexa Fluor 594 [1:500; for HA]) for three hours. Afterward, sections were rinsed three times (10 min each). Next, the sections were mounted on microscope slides using PBS, and coverslipped using fluoromount (Diagnostic BioSystems, Pleasanton, CA). Images were obtained using a Zeiss AXIO Imager M2. The following suppliers were used for the above materials: NDS (EMD Millipore, Billerica, MA), Triton X (Sigma-Aldrich, St. Louis, MO), mouse anti-TH (ImmunoStar, Hudson, WI), rabbit anti-HA (Cell Signaling Technology, Danvers, MA), donkey anti-mouse IgG (H+L) Alexa Fluor 488 (Thermo Fisher Scientific/Invitrogen, Waltham, MA), donkey anti-rabbit IgG (H+L) Alexa Fluor 594 (Thermo Fisher Scientific/Invitrogen, Waltham, MA).

Statistics

We analyzed the data with conventional parametric statistics (StatView, SAS Institute). Two-way analysis of variance (ANOVA) and repeated-measures ANOVA were used to assess general main effects and interactions ($\alpha = 0.05$). Results are shown as mean ± SEM.

SI Figures

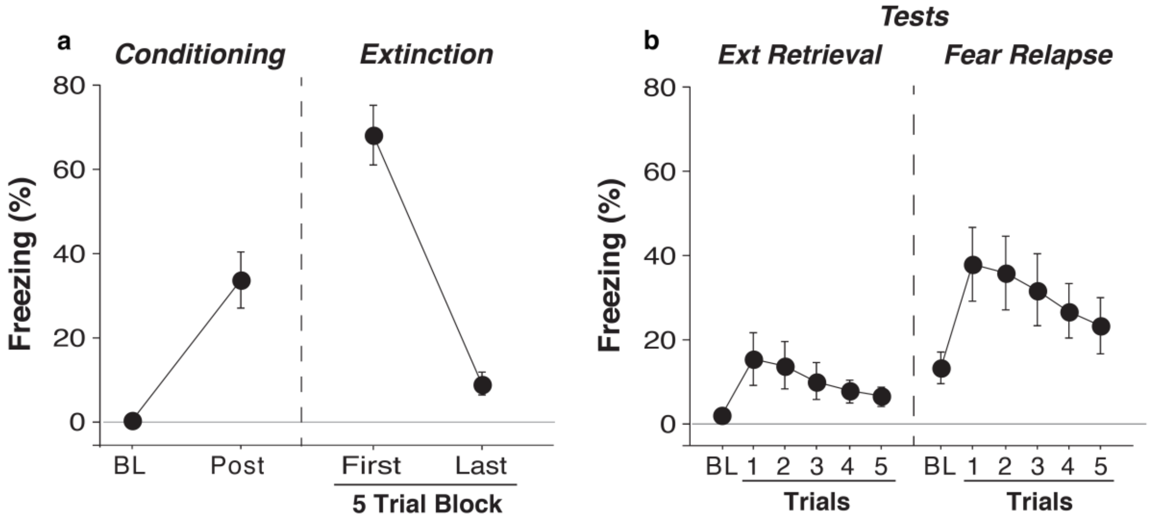

Figure S1. CS presentation outside the extinction context produces fear relapse. **a)** Percentage of freezing (mean ± SEM) from the conditioning and extinction sessions. Freezing is averaged across CSs and ITIs for all trial blocks in each session. All rats showed an increase in freezing behavior following conditioning [main effect of time, $F(1, 11) = 25.35$, $p < 0.001$]. The following two days rats showed a reduction in CS-evoked freezing behavior throughout extinction [main effect of time, $F(1, 11) = 44.05$, $p < 0.0001$]. **b)** Rats next underwent a dual retrieval-relapse test session. Rats showed low CS-evoked freezing in the retrieval context relative to the relapse context. The baseline freezing and the trial x trial data for the test session further illustrate that CS-evoked freezing was higher in relapse than in extinction retrieval [main effect of test, $F(1, 11) = 17.67$, $p < 0.01$].

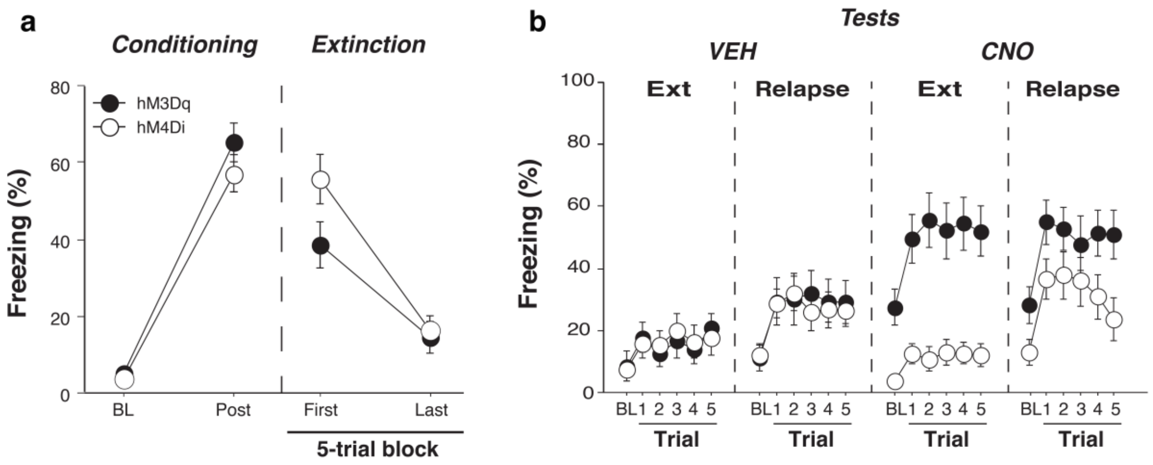

Figure S2. LC-NE activation produces fear relapse. **a)** Percentage of freezing (mean ± SEM); freezing is averaged across CSs and ITIs for all trial blocks in each session. Rats expressing either hM3Dq or hM4Di in the LC conditioned similarly as evidenced by an increase in freezing behavior from the baseline period to the post-shock period [main effect of time, $F(1, 40) = 285.61$, $p < 0.0001$]. Likewise, both groups extinguished fear to the CS over the course of the extinction sessions [main effect of time, $F(1, 40) = 64.62$, $p < 0.0001$]. After extinction, the animals received within-subject retention tests to the CS in either the extinction context or a familiar, alternate relapse context after administration of either VEH or CNO. **b)** The 3 min baseline (BL) freezing is shown as well as the freezing for each trial. While CNO produced a nonspecific increase in baseline (BL) freezing prior to CS onset (in the hM3Dq group) this does not account for the differences in CS-evoked freezing [drug x test x virus interaction, $F(1, 40) = 12.68$, $p < 0.001$].

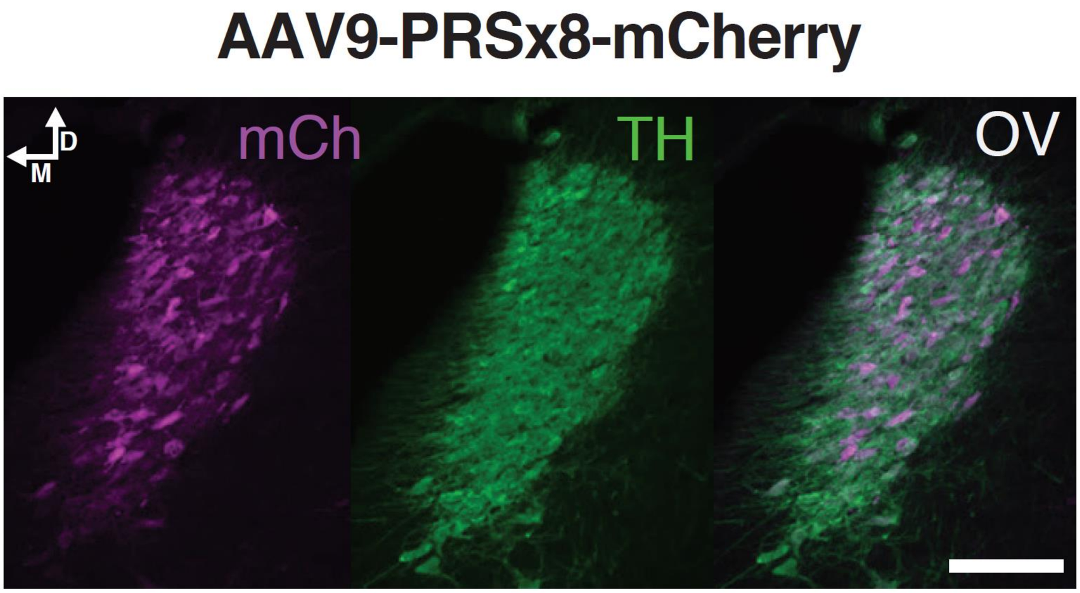

Figure S3. Control viral expression is restricted to the locus coeruleus. Immunohistochemical localization (OV, overlay) of the blank mCherry control virus (mCh, purple) in tyrosine hydroxylase-positive neurons (TH, green) in the LC. Scale bar = 100 um

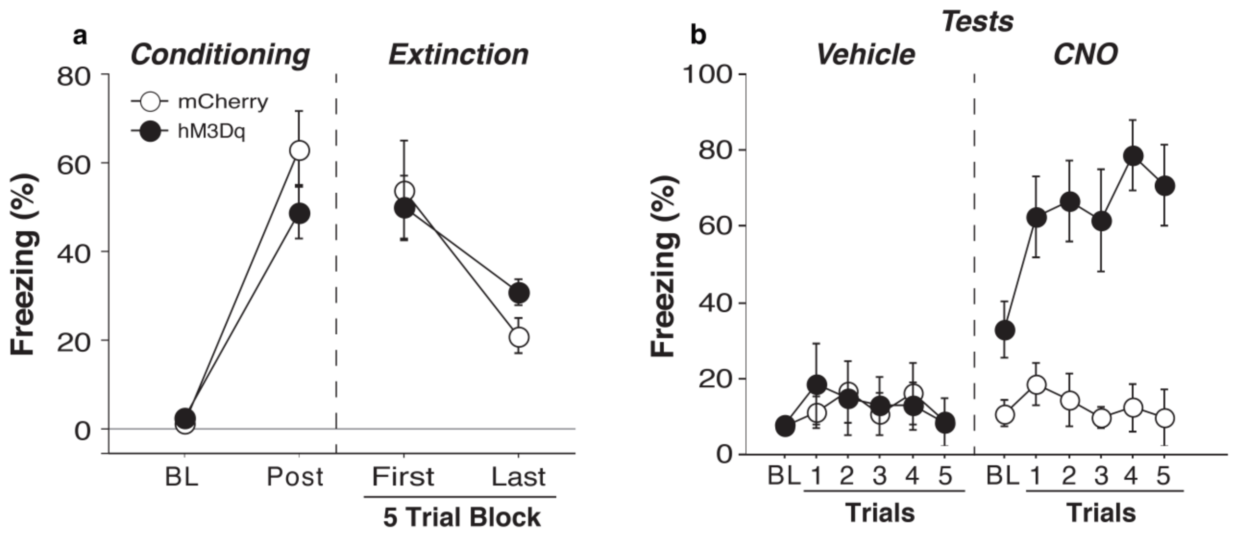

Figure S4. LC-NE induced fear relapse is independent of increases in baseline (pre-CS) freezing. **a)** Percentage of freezing (mean ± SEM) from the conditioning and extinction sessions. Animals expressing either hM3Dq or a blank mCherry virus in the LC showed similar increases in freezing from the pre-conditioning baseline period [main effect of time, $F(1,11) = 90.73$, $p < 0.0001$]. Both groups extinguished at a similar rate [main effect of time, $F(1,11) = 13.59$, $p < 0.01$]. **b)** The 3 min baseline (BL) freezing is shown as well as the freezing for each trial for the extinction retrieval sessions (each rat underwent one session after VEH or CNO administration). While CNO produced a nonspecific increase in baseline freezing in rats expressing hM3Dq, this does not account for the observed differences in CS-evoked freezing [time x drug x virus interaction, $F(5,55) = 3.53$, $p < 0.01$].

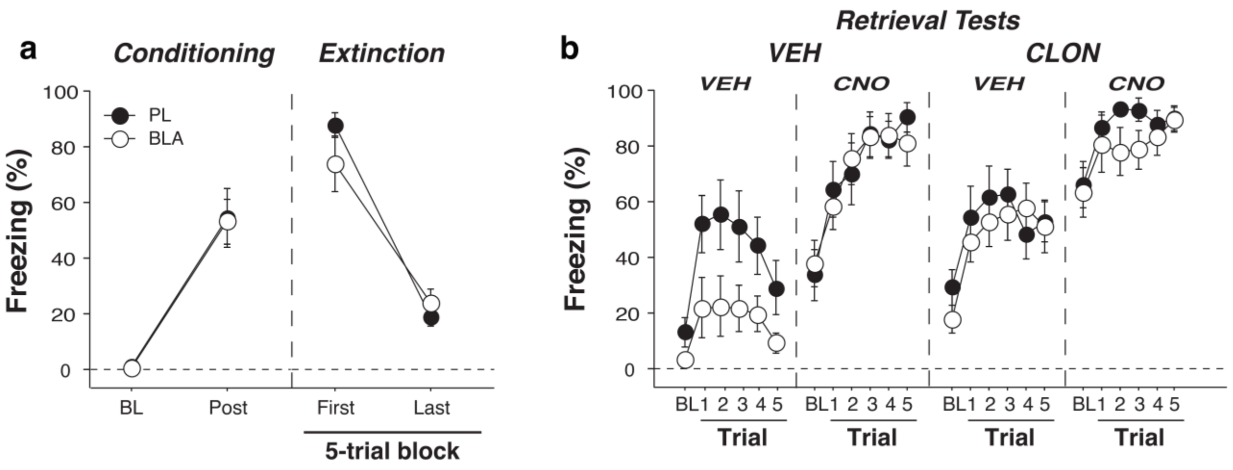

Figure S5. Local infusions of clonidine into either PL or the BLA block CNO-induced fear relapse independent of increases in baseline (pre-CS) freezing. **a)** Percentage of freezing (mean ± SEM) from the conditioning and extinction sessions split by brain region. Animals from both groups showed a similar increase in conditioned freezing from the baseline to the post conditioning period [main effect of time, $F(1,18) = 65.11$, $p < 0.0001$]. Both groups extinguished at a similar rate [main effect of time, $F(1,18) = 106.35$, $p < 0.0001$]. **b)** The 3-min baseline (BL) freezing is shown as well as the freezing for each trial for the extinction retrieval sessions (each rat underwent four total retrieval sessions). We replicate our finding where CNO-induced increases in LC-NE produce non-specific effects on baseline freezing, but this does not account for the differences in CS-evoked freezing. Local infusions of clonidine (alpha2-noradrenergic receptor agonist) into either the PL or BLA limit CS-evoked freezing in the presence of CNO (relative to nonspecific effects on baseline freeze) [systemic drug x local infusion x time interaction, $F(5, 90) = 5.11$, $p < 0.001$].

References

1. Vazey EM, Aston-Jones G (2014) Designer receptor manipulations reveal a role of the locus coeruleus noradrenergic system in isoflurane general anesthesia. *Proc Natl Acad Sci USA* 111(10):3859–3864.

2. Giustino TF, et al. (2017) β-Adrenoceptor Blockade in the Basolateral Amygdala, But Not the Medial Prefrontal Cortex, Rescues the Immediate Extinction Deficit. *Neuropsychopharmacology* 42(13):2537–2544.

3. Schulz B, Fendt M, Schnitzler H-U (2002) Clonidine injections into the lateral nucleus of the amygdala block acquisition and expression of fear-potentiated startle. *Eur J Neurosci* 15(1):151–157.

4. Holmes NM, et al. (2017) α2-adrenoceptor-mediated inhibition in the central amygdala blocks fear-conditioning. *Sci Rep* 7(1):11712.
